# Genetic Structure and Core Collection of Olive Germplasm from Albania Revealed by Microsatellite Markers

**DOI:** 10.3390/genes12020256

**Published:** 2021-02-10

**Authors:** Aida Dervishi, Jernej Jakše, Hairi Ismaili, Branka Javornik, Nataša Štajner

**Affiliations:** 1 Department of Biotechnology, Faculty of Natural Sciences, University of Tirana, Blv Zog I, 25/1, 1001 Tirana, Albania; aida.dervishi@fshn.edu.al; 2 Department of Agronomy, Biotechnical Faculty, University of Ljubljana, 1000 Ljubljana, Slovenia; jernej.jakse@bf.uni-lj.si (J.J.); branka.javornik@bf.uni-lj.si (B.J.); 3 Gene Bank, Genetic Resources Centre, University of Agriculture, 1020 Tirana, Albania; hairiismaili@yahoo.fr

**Keywords:** *Olea europaea*, genetic variability, microsatellite, EST-SSR, core collection

## Abstract

Olive is considered one of the oldest and the most important cultivated fruit trees in Albania. In the present study, the genetic diversity and structure of Albanian olive germplasm is represented by a set of 194 olive genotypes collected in-situ in their natural ecosystems and in the ex-situ collection. The study was conducted using 26 microsatellite markers (14 genomic SSR and 12 Expressed Sequence Tag microsatellites). The identity analysis revealed 183 unique genotypes. Genetic distance-based and model-based Bayesian analyses were used to investigate the genetic diversity, relatedness, and the partitioning of the genetic variability among the Albanian olive germplasm. The genetic distance-based analysis grouped olives into 12 clusters, with an average similarity of 50.9%. Albanian native olives clustered in one main group separated from introduced foreign cultivars, which was also supported by Principal Coordinate Analysis (PCoA) and model-based methods. A core collection of 57 genotypes representing all allelic richness found in Albanian germplasm was developed for the first time. Herein, we report the first extended genetic characterization and structure of olive germplasm in Albania. The findings suggest that Albanian olive germplasm is a unique gene pool and provides an interesting genetic basis for breeding programs.

## 1. Introduction

Olive (*Olea europaea* L.) is one of the most important and oldest cultivated plants in the Mediterranean Basin and it is an emblematic tree crop, even in Albania. Olive has been grown in Albania since ancient times due to the favorable climatic and ecological conditions. The cultivation of olive in Albania is thought to have begun approximately at the same time as in neighboring regions. The presence of olives in Albania has been historically demonstrated by records of olive oil trade by 300–150 BC, finds at archeological sites of olive mills dating back to the 4th Century BC [1], olive stones, and decorated amphorae’s with olive trees [2]. The enormous number of ancient cultivars and oleasters with an estimated age of up to 3000 years provides a living proof of olive antiquity in Albania [1]. The olive orchards are mainly located at hilly zones and on the western coast and central region, and a few olive orchards which constitute a very interesting pool regarding their genotypes’ adaption to colder climate are found in the northern part of Albania. The co-existence of cultivated olives with their wild relatives, the oleasters, in Mediterranean countries, has been inferred from archeological and paleobotanical findings [3]. In some areas, cultivars and feral-wild olives live together within a radius of a few meters and they are not easily distinguished [4]. The diversity of feral populations, due to the derivation of a new seedling by sexual reproduction, grown without any agricultural aid, could give rise to varieties with superior traits for cultivation [4]. Furthermore, the oleasters could contribute to the explanation of domestication processes [5]. Presumed crosses between wild and cultivated forms may have led to new cultivars around the Mediterranean countries [6]. In Albania, feral and wild olives (oleasters) have also been found in isolated areas and in association with cultivated olives, for which they are thought to be pollinators, and their estimated age ranges from 200 to 1000 years [7] Frezzoti, 1930, cited by Reference [8].

Due to the increased demand for olive oil, as well as table olives, the Albanian government promoted an increase of olive trees to be planted in the country, up to 25 million olive trees over several years [9]. The planting campaign is still going on and at present, there are ten million olive trees recorded [10], stressing the need for the correct characterization to ensure preserving the main cultivars and safeguarding minor olive genotypes, avoiding genetic erosion. Molecular marker methods have undoubted advantages, including microsatellites, which have proven to be powerful tools for the identity, parentage, and kinship analysis of a wide range of plant species. Microsatellite markers have been used in various studies on olives, such as identification purposes, relationship establishment, population structure analyses, core collections sorting, etc. Microsatellite markers have been applied successfully in the assessment of genetic diversity and relationships among wild olives [11], among cultivated and wild olives [5,12,13], as well as within native and introduced cultivated olives [14]. The usefulness of microsatellite markers has been confirmed as a powerful tool for assessment of genetic relationships among cultivated olives of different regions by assigning cultivars according to their area of origin [15,16,17]. Microsatellites are also preferentially used in inferring the structure of populations [12,13,18] and establishing core collections for the conservation and the optimal management and use of olive genetic resources [18,19,20,21]. Furthermore, projects on expressed sequence tags (EST) of olives [22,23,24,25] offered the possibility of obtaining new microsatellites with less effort and costs. The development of expressed sequence tag-SSRs(EST-SSR) markers in olives has been reported in several studies [26,27,28,29,30] and they were successfully used to assess variation in coding regions of the genome. In this scenario, the combination of the genomic microsatellites (gSSR) and EST-SSR molecular markers will provide a powerful tool for assessment of genetic variability in noncoding and in the coding regions.

Albanian olive germplasm evaluation has to date been traditionally characterized and identified by morphological and agronomical traits, which are known to be influenced by environmental factors and the developmental stage of the plant. The most recent olive catalogue, based on morphological descriptors and oil content, describes 55 autochthonous olive genotypes, of which 45 are cultivated olives and 10 are oleasters [7]. The molecular studies conducted so far included a limited number of Albanian olives analyzed by means of randomly amplified polymorphic DNA (RAPD) markers [8] and by nine SSRs [10].

The aim of the study was the evaluation of the genetic diversity, assessment of genetic relationships of olive genotypes, and the variability among different geographical and breeding groups of olives and establishing core collection. The molecular data provides for the first time a broad inventory of olive genetic resources in Albania.

## 2. Materials and Methods

### 2.1. Plant Materials

A set of 194 samples of olive accessions (*O. europaea* ) were collected in situ and in the ex-situ collection in Albania, encompassing the representatives of local olive cultivars (130), cultivars of foreign origin (45) that have been introduced (mainly from Italy), and oleasters (19), which are used mainly as rootstocks and are pollen donors for many cultivated olive cultivars. A group of 74 olive trees represent very old plants, with an estimated age of 500–3000 years, maintained in situ in their natural ecosystem at olive growing regions throughout Albania. The ancient olive genotypes were previously morphologically characterized and selected to construct an Albanian in situ olive germplasm collection under the auspices of the Gene Bank of Albania.

### 2.2. Microsatellite Genotyping

Total genomic DNA was extracted from young leaf tissue using the CTAB (cetyltrimethylamonium bromide) method [31]. DNA concentration was quantified based on fluorescent detection, on a DyNAQuantTM 200 fluorometer (Amersham Biosciences, Chicago, IL, USA), according to the manufacturer’s instructions. The sample set was genotyped with a set of 26 microsatellite markers, of which 14 were genomic SSRs [32,33,34,35] and 12 were EST-SSRs [30].

The SSR analysis was performed following the approach of Schuelke [36], by appending a fluorescently labelled M13 tail to the forward primers. The detailed protocol is described in Dervishi et al. [30]. Microsatellite loci were amplified using a touchdown PCR protocol, by using the thermal profile described in Dervishi et al. [30].

### 2.3. Data Analysis

For each locus, particular attention was paid to genotyping errors, such as potential allele dropout, which can lead to a decrease in sample heterozygosity and to stuttering patterns, which can hide the true allele peak. The resulting low-frequency alleles occurring ≤ 5 times were double checked on the original pherogram and any genotyping errors were corrected accordingly. The number of alleles per locus (No), the observed and expected heterozygosity (Ho and He), Hardy-Weinberg equilibrium (HW), polymorphic information content (PIC), and frequency of null alleles (F_null_) were calculated with Cervus 3.0 software [37] upon the set of resulted unique genotypes. Ne (effective number of alleles) and the frequency of alleles (Freq ≤ 5) were calculated for each olive group defined by geographic origin and breeding using GenAlEx v 6.501 [38,39]. Genetic relatedness was evaluated by estimating DICE’s similarity index [40], implemented in the statistical software NTSYS v 2.2 [41]. Cluster analysis was performed based on the unweighted pair group method with an arithmetic mean (UPGMA) algorithm. Principal coordinate analysis (PCoA) was performed based on a pairwise, individual-by-individual genetic distance matrix calculated for codominant data and also by intercomparison of the different olive groups/populations defined by their different breeding (cultivated and foreign olive genotypes, oleasters), geographic origin/main area of distribution (Ionic region and Adriatic region), and their product end-use (table olive vs. olive oil production).

Analysis of molecular variance (AMOVA) was carried out to determine the relative partitioning of the total genetic variation among and within different groups of olive genotypes by using GenAlEx 6.5 [38,39]. The significance of the Φ_PT_ index was tested by 9999 permutations.

The model-based Bayesian method in STRUCTURE version 2.3.4 software [42] was applied to multi-locus microsatellite data to infer the genetic structure. STRUCTURE HARVESTER version 0.6.93 [43] was used for visualizing STRUCTURE output and the Evanno method for detecting the number of clusters of individuals [44]. Ten runs of STRUCTURE were performed by setting the number of clusters (K) ranging from 1 to 10. Each run consisted of a burn-in period of 200,000 steps followed by 10^6^ MCMC (Monte Carlo Markov Chain) replicates, assuming an admixture model and correlated allele frequencies.

Finally, we assembled a core collection from the large database of individual molecular profiles of our sample set, using the M strategy [45,46], applied with CoreFinder software. The algorithm for inferring the core collection was obtained heuristically.

## 3. Results

### 3.1. Genetic Diversity

The genetic variation among 183 unique olive genotypes identified by identity analysis was estimated using 26 SSR markers. The list of unique genotypes is presented in the Supplementary Table S1. A total of 203 alleles were amplified across all samples. The number of alleles per locus ranged from 2 (SiBi 03 and SiBi 11) to 19 (DCA09), with a mean of 7.8 alleles, revealing a high level of variability in our sample set. The observed heterozygosity value (Ho) ranged from 0.357 (SiBi 03) to 0.939 (DCA03), with a mean of 0.744, while the expected heterozygosity (He) ranged from 0.294 (SiBi 03) to 0.873 (DCA09), with a mean of 0.678. The observed heterozygosity showed higher values than the expected heterozygosity across 19 loci, while it showed a slightly lower value than expected heterozygosity at 7 loci out of 26, (DCA05, DCA09, DCA11, DCA16, GAPU59, UDO24, and SNB19). Polymorphic information content (PIC) ranged from 0.250 (SiBi 03) to 0.859 (DCA09) respectively, with an average of 0.630. As we previously reported, high PIC values (>0.5) were found in 73% of loci, and we classified these loci as highly informative. Forty-six percent of loci (DCA03, DCA09, DCA16, DCA18, GAPU59, GAPU71B, GAPU101, UDO24, EMO90. SNB03, SNB11, SNB14) showed PIC > 0.7 and could be classified as potential markers for genetic mapping. The minimum of eleven markers were identified by AMaCAID script as sufficient to differentiate among 183 genotypes [30].

The unique Albanian genotypes (183) were compared with 80 olive genotypes from 11 olive growing countries (deposited in the World Olive Database) at ten microsatellite loci and with 19 olive cultivars from Slovenian collection at 26 loci. The comparative analysis did not find cases of synonymy among compared microsatellite profiles.

### 3.2. Genetic Diversity of Oleasters and Olive Cultivars

The parameters of diversity calculated for 183 unique olive genotypes (comprised 18 oleaster genotypes, 120 native, and 45 foreign olive cultivars) are presented in the Supplementary Table S2. The average number of alleles per loci obtained for 120 native cultivated trees (6.92) and for the foreign cultivars (6.2) resulted higher than the average number of alleles amplified in oleaster genotypes (5.31), while the mean number of effective alleles (Ne) was in the same range among analyzed olive groups, ranging from 3.28 in native cultivars, 3.51 in oleasters, and 3.72 in foreign cultivars. In total, 46 private (unique) alleles were detected in three olive groups. The highest number of private alleles was found in native cultivated genotypes, 24 (52%), whereas 13 (28.3%) private alleles belonged to foreign olive cultivars and only 9 (19.6%) were observed in oleaster genotypes. The mean expected heterozygosity resulted higher in oleaster genotypes (He = 0. 692) than in cultivated olives (He = 0.657), whereas the mean observed heterozygosity resulted higher in cultivated olives (Ho = 0.760) than in oleaster genotypes (Ho = 0.745) (Table 1).

### 3.3. Genetic Relatedness

The clustering analysis highlighted 12 groups of olive genotypes with mean similarity of 0.509. Each of the main groups subdivides into several small clusters consisting of related genotypes. In many cases, the high level of genetic similarity between genotypes of the same subcluster (>85%) was observed. The genetic relationships among 183 olive genotypes were visualized by a dendrogram, shown in Supplementary Figure S1 and Table S2. The native Albanian olive genotypes were tightly clustered with each other in three clusters (cluster IV, V, and VI) containing 99 (95%) Albanian genotypes and 5 (5%) foreign cultivars (4 Italian and 1 USA).The rest of the Albanian genotypes were dispersed within other clusters admixed with some foreign cultivars. While in clusters II and IX, the majority of foreign cultivars were observed, 15 (75%) and 13 (62%), respectively. There is a clear structuring of the variability relative to the geographic main distribution area of Albanian native genotypes. Genotypes from central and northern regions (Adriatic region) tend to be grouped in distinct clusters from those from southern Albania (Ionic region). The Adriatic genotypes grouped mainly in three clusters, the cluster IV (subcluster I): 93.6% (44 genotypes out of 47), cluster V: 90% (18 genotypes out 20), and cluster VI: 83.3% (10 Adriatic genotypes out of 12). The majority of Ionic genotypes, on the other hand, were in cluster V (subcluster II): 80% (20 out of 25), and cluster XII: 71.4% (5 out of 7 cultivars).

The majority of genotypes used as table olives and dual use were dispersed in clusters I, II, and IV (subcluster II). Most genotypes used for oil production were dispersed in clusters IV (subcluster I), V, VI, and XII. Only native genotypes, especially those from the central region, showed fairly good clustering according to their fruit end-use. Only five oleasters out of 18 in cluster VI were subclustered distinctly. Most of the oleasters (13 out of 18) clustered together with Albanian cultivated forms, especially with foreign cultivars, in the different groups (Supplementary Figure S1), but five of them clustered separately (Supplementary Figure S1—cluster VI), suggesting a genuine originality of these oleaster genotypes. No clear distinction of cultivated olives and oleasters was observed in analysis of relatedness.

### 3.4. Genetic Structure and Principal Coordinate Analysis

**Figure 1:**
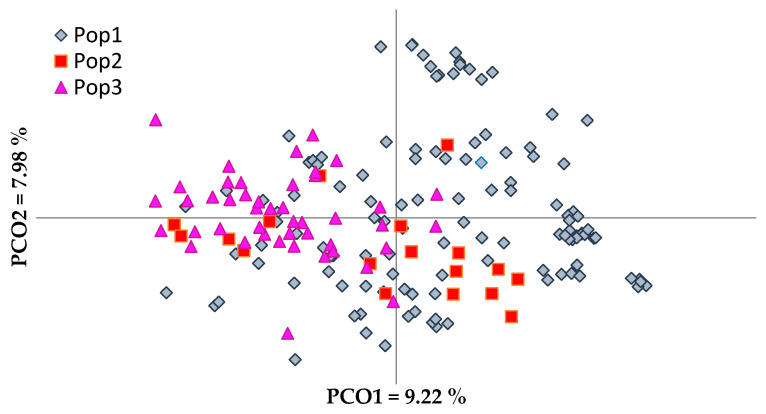
Principal coordinate analysis (PCoA) of olive genotypes based on their origin and breeding. Pop 1—Native cultivars, Pop 2—Oleasters, Pop 3—Foreign imported cultivars.

**Figure 2:**
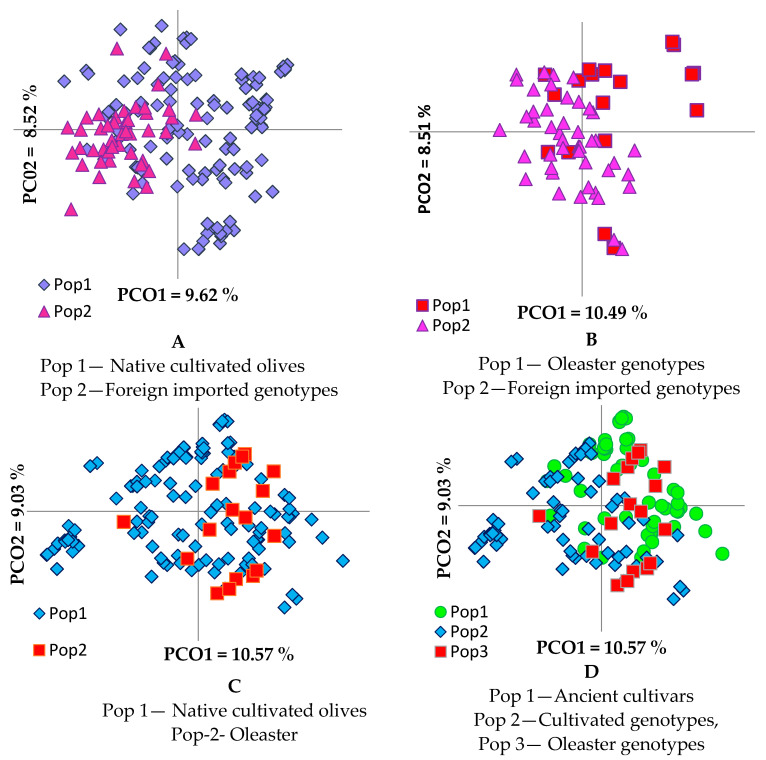
Principal coordinate analysis of olive genotypes based on their origin (**A**,**B**) and breeding (**C**,**D**).

Principal coordinate analysis (PCoA), available in GenAlEx v 6.501 software [38,39], was performed on a genetic distance-based matrix on the complete dataset of 183 unique olive genotypes, and on specific groups of olive genotypes defined by their origin/main area of distribution, breeding (cultivars vs. oleasters), and by their product end-use to graphically present the relationship between individuals/groups of olives, and determines whether partitioning into these groups is supported by genetic variation. A high level of overlapping was shown in the PCoA scatter plot of the analysis of the entire dataset of the 183 olive genotypes. PCoA analysis was also performed for different groups, native vs. foreign genotypes and Ionic region vs. Adriatic region genotypes, as well as foreign cultivars vs. native cultivars and oleasters (Figure 1 and Figure 2).

The PCoA explained a total variation of 23.21% (Figure 1). The first principal coordinate accounted for 9.22% of the total variation and allowed the discrimination of the majority of foreign olive cultivars from native olive cultivars and oleasters. The second coordinate, which accounted for 7.98% of the total variation, differentiated the majority of the oleasters. There was no clear separation of oleasters from native cultivated cultivars or foreign ones. The same was observed in the phenogram (Supplementary Figure S1), in which some oleasters were closely clustered with cultivated olive cultivars.

PCoA in pairwise comparison of olive groups (Figure 2) revealed a clear differentiation between foreign cultivars and the group of oleasters (Figure 2B), while the differentiation was not as clear between the groups of foreign cultivars and native cultivated olives (Figure 2A). Oleaster genotypes were admixed much more with the ancient than with the current olive cultivars (Figure 2C,D). The analysis of molecular variance (AMOVA) performed to evaluate the partitioning of molecular variance among native cultivars, oleasters, and foreign olive cultivars, showed that 93% of variation is due to genetic variation within groups, with only 7% of genetic variance being observed among groups, indicating the high heterozygous nature and mixed genetic structure of the olive genotypes [47]. The calculated PhiPT (analogue of Fst index) (0.074) was significant, *p* < 0.001, indicating low genetic differentiation among groups. The *p*-values were calculated for a random permutation test of 9999 permutations.

Based on a pairwise population matrix of Nei’s genetic distances (Supplementary Table S3), the highest genetic distance (GD) was observed between foreign cultivars and oleasters (0.127) and the lowest between native and foreign cultivars (0.092). Oleasters were shown to be closer to native cultivars (GD = 0.106) than foreign cultivars (GD = 0.127).

**Figure 3:**
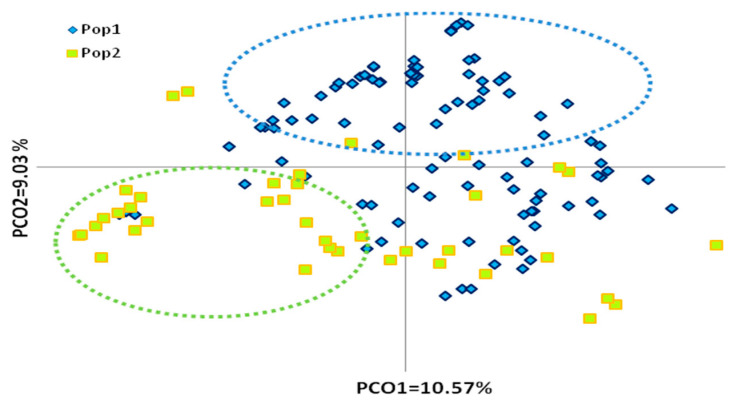
PCoA plot of 183 native olive genotypes, defined according to their main geographical area of distribution/origin (Adriatic vs. Ionic region). Pop 1—Genotypes distributed at Adriatic region, Pop 2—Genotypes distributed at Ionic region.

Analysis of principal coordinates based on assignment of two geographical groups according to their main area of distribution/origin (Ionic vs. Adriatic region) explained a total of 26.30% of genetic variation. The first two principal coordinates accounted for 10.57 and 9.03 of variation, respectively (Figure 3). The slight overlapping of olive genotypes did not allow them to be divided into two clear groups, although the differentiation of genotypes from Ionic and Adriatic regions was obvious because the majority of them were separated by the second principle coordinate, confirming previous results that were based on morphological characters [1]. In addition, analysis of molecular variance (AMOVA) performed to estimate the level of differentiation between these two groups (Ionic and Adriatic) revealed higher values of variation between individuals within each group (92%) than between these two groups (8%). The calculated PhiPT (0.081) was weakly significant (*p* < 0.05). Nei’s genetic distance between the group of genotypes of the Ionic region and that of the Adriatic region was 9.4%.

**Figure 4:**
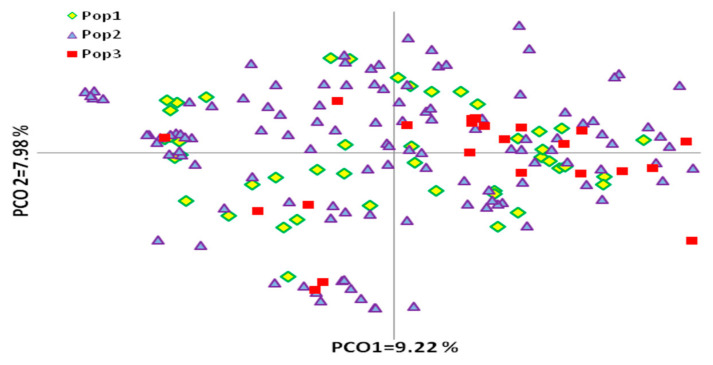
PCoA plot of 183 olive genotypes defined according to their fruit end-use. Pop 1—olive cultivars used for double purposes, Pop 2—cultivars used for oil production, Pop 3—cultivars used as table or canned olives production.

The links among these three groups of olive genotypes based on their product end-use was evaluated by PCoA (Figure 4). The olive genotypes were not separated into groups according to their end-use in the PCoA diagram. Principal coordinates explained 23.21% of genetic variation: the first and second coordinates revealed 9.22% and 7.88% of variation, respectively. AMOVA revealed that most genetic diversity was attributable to variability within olive groups (96%) rather than between groups (4%) of different products’ end-use. The calculated PhiPT = 0.045 for all olive groups used for oil, table, or dual use was significant (*p* < 0.05). The admixture observed in the principal coordinate analysis suggests that the abundant diversity existing in olives cannot be differentiated based on the end-use of the product.

PCoA indicated that, despite slight overlapping, the olive genotypes showed grouping according to their origin and their area of distribution. The differentiation of genotypes according to their fruit end-use obtained in UPGMA analysis was not supported in the PCoA analysis.

**Figure 5:**
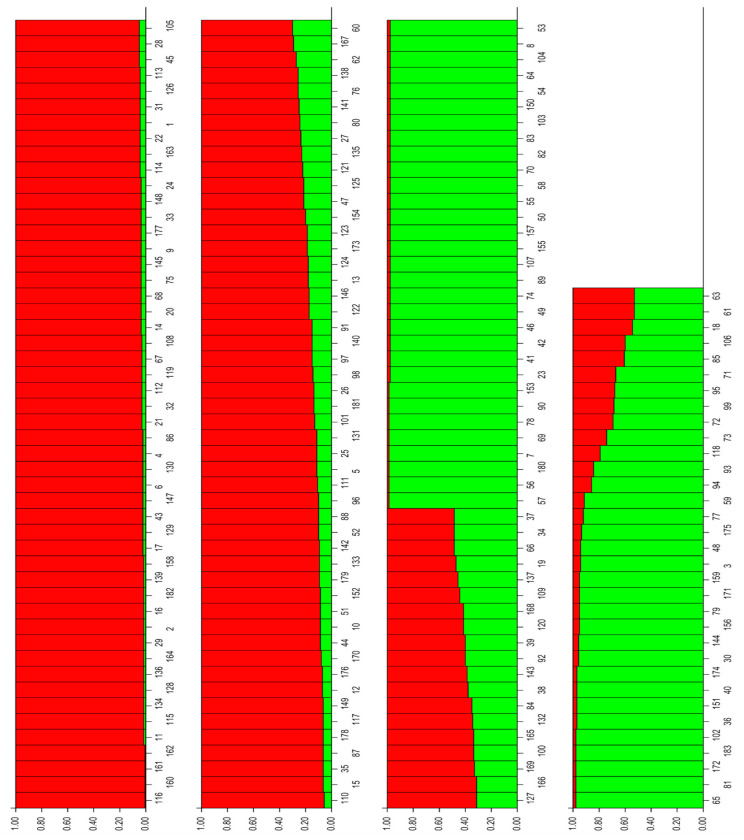
Inference of population structure based on microsatellite data and Bayesian simulation.

A model-based Bayesian approach was performed to infer the genetic structure of individuals under K = 2 clusters, which, as estimated by Evanno’s ΔKs statistics [44], resulted in the best fit to the model for olive genetic structuring. The proportion of its genome derived from different clusters was estimated. The plot of the mean log-likelihood values (Ln(K) ± SD) averaged over 10 iterations and Evanno test for delta K are shown in Supplementary Figure S2. Genotypes were assigned to a cluster when 85% or more of their inferred genomes belonged to the cluster, with genotypes having a lower percentage being considered to be admixed [48]. Using a threshold of >85% for the designation of group representatives, 81 genotypes were assigned to group A (red bars) and 53 genotypes to group B (green bars), and the remaining 49 genotypes (26.7%) were assigned as admixed genotypes (Figure 5, Table S4).

Hierarchical levels of population structure could hardly be recognized. Both groups, A and B, are of mixed usage origin (oil, table, or dual use) and no well-assigned population designations could therefore be recognized. We observed only weak differentiation, as in group A (red color), containing 81 olive genotypes, there is 9 oleasters, the majority of foreign imported olive cultivars (39), and some native cultivars (33), while group B (green color) is composed of 53 native cultivars and lacks the oleaster genotypes and foreign imported cultivars. This kind of differentiation was also observed in PCoA and AMOVA, where only 7% of variation was observed among native cultivars, oleasters, and foreign cultivars, indicating a mixed genetic structure of the olive genotypes.

The overall assignment of the samples to each of two clusters were 58.9% and 41.1% for cluster A and B, respectively. Average distances (expected heterozygosity) among individuals within a cluster were 0.708 and 0.559 for clusters A and B, respectively. The low-differentiation structure (K = 2) detected within our analyzed set of samples may be due to complex relationships among the olive cultivars.

### 3.5. Sorting Out a Core Collection

**Figure 6:**
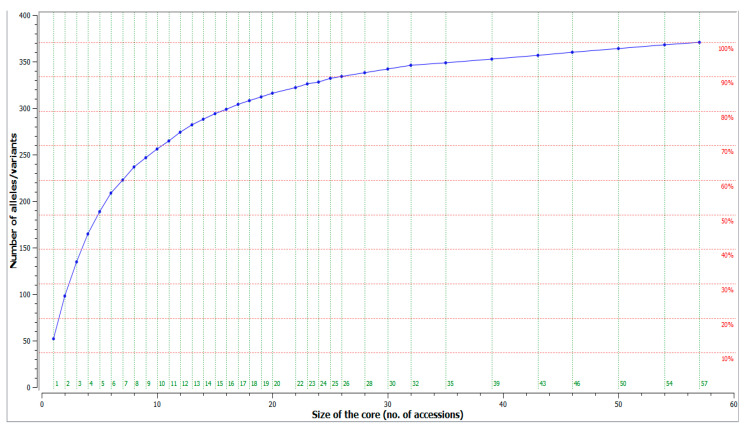
Genetic diversity as a function of the number of accessions included in the core collection.

The dataset of microsatellite profiles of 183 unique olive cultivars for 26 microsatellite loci was used to construct an olive core collection, using the M strategy [45,46], implemented in CoreFinder software. A core collection was herein assembled for each of the olive germplasm collections maintained ex situ, in situ, and for the overall Albanian olive germplasm, aiming to represent the entire genetic diversity identified in this study (Figure 6).

The entire allelic richness of the actual ex situ core collection (117 accessions) may be represented by a minimum number of 45 accessions (38%), while the diversity of the in situ collection (66 accessions) could be represented by 32 accessions (48%).

However, for better management of the two olive collections in Albania and the preservation of the germplasm in a thorough and efficient way, the collections were unified to sort out a core collection of all Albanian olive germplasm. CoreFinder analysis showed that 203 alleles identified by characterization of 183 unique genotypes with 26 microsatellite loci could be represented by a core collection of 57 accessions (31%) of all genotypes (Supplementary Table S5).

## 4. Discussion

### 4.1. Olive Germplasm Genetic Diversity

Herein, this study examined genetic diversity and structure of Albanian olive germplasm, consisting of native and introduced foreign cultivated olives and of oleasters. In total, 26 microsatellites including 12 EST-SSRs were employed in this study. The analyses revealed in total 203 alleles, with an average 7.8 alleles per locus, revealing a high level of variability within a sample set. The obtained average number of alleles per locus is in the same range as that reported for: 489 Italian olive varieties (7.6) [21], 84 Tunisian accessions (8) [49], 30 cultivars from Southern Italy (8.8) [50], 48 cultivars from the Iranian olive collection (9) [51], 108 accessions from the Davis collection, USA (9.93) [52], higher than that reported for 10 Turkish cultivars (4.57) [53], 211 Italian olive cultivars from Southern Italy (6.82) [16], 10 Iranian cultivars (5.6) [54], 60 Brazilian accessions (6) [47], 27 accessions from Istria (6.75) [55], 19 cultivars from the Slovenian olive collection (6.8) [56], 33 Tunisian accessions (7) [57], and lower than that reported for 142 Italian cultivars from Emilia-Romagna (10.2) [58], 73 olive trees, including wild, cultivated, and ancient trees of Sardinia, Italy (10.46) [13], 46 Portugese cultivars (11) [59], 77 olive cultivars from the two olive collections, World Olive Germplasm Bank (WOGB), Spain, and Agricultural Research Council-Olive Growing and Oil Industry Research Centre collection (CRA-OLI) (12.2) [60], 505 accessions derived from 14 olive growing countries in the OWGB germplasm of Marrakech (12.5) [18], 118 cultivars from the main Mediterranean olive-cultivating countries (13.2) [15], 30 wild and 104 cultivated ancient olive trees of the Andalusian region, Spain (13.64) [61], 104 Greek accessions (13.5) [62], and 158 samples of wild and cultivated olives from three olive growing areas in Spain (16.5) [12]. The high number of alleles obtained in some studies may be due to the use of a large amount of highly diversified plant material [60,63], as well as the high number of samples employed in the analysis. The lower mean number of alleles observed in our olive genotypes could be explained by the low degree of polymorphism revealed by some of the EST-SSR used, which is lower than the degree of polymorphism detected by genomic SSR used in previously reported studies. Nevertheless, the latter gave better quality of allelic pattern and were still sufficiently informative and showed slightly higher level of heterozygosity than genomic SSRs [30].

The observed heterozygosity resulted higher than the expected one at the majority of loci (19), while we only observed lower observed heterozygosity on 7 loci compared to the expected heterozygosity. This observed heterozygosity deficiency may be related to the presence of null alleles, whose frequency values were positive at six of these loci [30]. However, the expected heterozygosity in our study showed the mean of 0.678. This value was similar to those reported in other studies, such as Fendri et al. [49] (0.680) and Diez et al. [61] (0.698). The overall heterozygosity values in our olive genotypes was high, thus indicating the presence of broad genetic diversity, which is explained by a high selection of cultivars showing traits of interest, such as the size of the fruit or oil content. The presence of null alleles favors an increase of homozygosity over heterozygosity, and the occurrence of null alleles has already been described for the same microsatellite primers by other authors [54,60,64,65].

The PIC value indicates the level of polymorphism information provided, as well as the usefulness of the microsatellite primers for genotyping, gene mapping, molecular breeding, and germplasm evaluation. In this sense, the most suitable loci for genetic characterization of the analyzed set of olive genotypes were DCA03, DCA09, DCA18, SNB03, and SNB11, which showed PIC values equal to or higher than 80% [30].

The comparative analysis of 183 unique genotypes with the allelic profiles of 99 olive genotypes belonging to 12 olive growing countries did not find cases of synonymy, supporting the hypothesis of an autochthonous origin of Albanian cultivars [7,8,66] and that the Albanian olive cultivars represent a unique gene pool.

### 4.2. Comparative Assessment of Genetic Diversity of Oleaster and Cultivated Olives

Botanists have reported two varieties of *O. europaea* subsp. *europaea*: the cultivated form var. *sativa* and wild form var. *sylvestris,* also called ‘oleaster’. Both varieties are diploid species (2n = 46) [67,68]. True oleaster has been found in forest or land with apparently no relation to a cultivated area, whereas feral forms are found around current orchards or in old deserted orchards [69]. Presumed crosses between wild and cultivated forms may have led to new cultivars around the Mediterranean countries [6]. However, the contribution of oleaster on the evolution of cultivated olive is still a widely debated issue, which relates to the distinction between real oleasters and feral plants derived from natural dissemination of cultivars, since these two forms may show a similar appearance when grown in the same ecological sites [70]. Several studies investigated genetic diversity and the relationship between cultivated and wild olives [5,12,13] by microsatellite markers. Assessing genetic diversity of olive germplasm is essential for its efficient utilization. Wild olive genotypes are considered as a source of genes related to the resistance to biotic and abiotic factors; as such, they constitute an important source for the breeding programs, especially for genes linked to the resistance to harsh environmental conditions that might be under upcoming climate change. Prior knowledge of genetic diversity of oleasters as well as their relatedness to cultivated olive genotypes provides efficient utilizations of these resources in Albania. Therefore, in this study, we assessed comparative genetic diversity among 18 oleaster genotypes collected in situ in old orchards, 120 native Albanian cultivars and 45 introduced foreign olive cultivars. Genetic diversity among the analyzed groups resulted in lower mean allele number in oleasters compared to native and foreign olive cultivars (Table 1). However, the number of alleles per locus detected in Albanian oleaster genotypes (5.1) was in the same range as that detected in 48 genotypes of wild olive in Spain (5.62) [11], but lower than the average number of alleles detected in a study of 21 wild olive individuals in the region of Sardinia (8) [13]. In agreement with the previous studies that compared genetic diversity in wild and native cultivated olive groups [12,13,18], the mean expected heterozygosity value was higher in oleaster genotypes than in cultivated olives, whereas the observed heterozygosity values were higher in cultivated olives than in oleaster genotypes. However, the level of heterozygosity shown by oleaster genotypes was similar to previous studies carried out in wild olives [11,13,63,71]. The lower diversity detected in oleaster genotypes compared to native cultivated olives in our study could be due to the low number of oleasters (18) in comparison to cultivated olives (120). It might also be due to the possibility that some of the sampled oleasters represent feral forms.

### 4.3. Genetic Relatedness

Based on the results of genetic relatedness, our sample set showed an average similarity of 0.509, which was higher than the average similarity found in olive cultivars from the Slovenian olive collection (0.26) [56] and within the range of mean similarity found in Tunisian olives (0.574) [72]. The native Albanian genotypes were highly related with each other in three clusters, comprising more than 95% of Albanian genotypes. High relatedness and a clear distinction of Albanian cultivars from foreign ones (Greek, Italian, and Turkish) was also observed by Belaj et al. [8], indicating their autochthonous origin.

The area of origin can be identified because of the climatic and pedological conditions under which populations experienced a secondary structuration of variability. Distinguishing among varieties belonging to different gene pools would enable selection of the most adapted cultivars for the new breeding policies for each geographically defined area [15]. The olive distribution area in Albania is divided into two main regions, based on the correlation of morphological diversity with the climatic conditions and geographical elements of these areas. The olive germplasm is thus distributed into the south–western area, under the influence of the Ionian Sea (Ionic region), and the central and northern areas under the influence of the Adriatic Sea (Adriatic region) [1]. There was a clear structuring of the variability observed relative to the geographic origin/main distribution area of Albanian native genotypes. Genotypes from central and northern regions (Adriatic region) tend to be grouped in distinct clusters from those from southern Albania (Ionic region), while the majority of Ionic genotypes, on the other hand, were clustered distinctly. The obtained clustering may also be due to selection pressure towards cultivars that are adapted to the different climatic conditions of these two regions [8].

Weak clustering in relation to the fruit end-use was observed in the overall sample set, in line with the findings of Abdelhamid et al. [57]. Only native genotypes, especially those from the central region, showed fairly good clustering according to their fruit end-use. Clustering in relation to the origin was observed for 5 out of 18 oleasters that were distinctly clustered, suggesting a genuine originality of these oleaster genotypes. The other oleasters clustered together with Albanian cultivated forms, especially with foreign cultivars, supporting two hypotheses: (1) oleasters grouped in the same cluster with cultivated forms may be feral olives and are showing high similarity with the cultivated genotypes from which they derive, or (2) according to the second hypothesis, this may be a case of clustering of true oleasters together with cultivated forms that derived from them. No clear distinction of cultivated olives and oleasters was observed, which is in accordance with previous studies [12], indicating the presence of feral forms in our sample set. However, the co-existence of true oleasters and feral forms should not be excluded. The analysis of a larger number of oleasters, including oleasters from neighboring countries, would contribute to a better understanding of origin and distribution in Albanian olives.

### 4.4. Genetic Structure

The model-based method, PCoA, and analysis of molecular variance showed a wide range of variability within our olive groups. The geographical grouping according to olive origin and area of distribution was confirmed, though not in a clear-cut way. There was weak differentiation of olive genotypes based on fruit end-use. No hierarchical differentiation based on olive end-use products was revealed by PCoA, either (Figure 4). This level of admixture is possibly also due to the make-up of the sample set, in which native and foreign cultivars and oleaster forms are included. The resulting overlapping of oleasters mostly with native cultivars, especially ancient ones, might be due to the age of ancient cultivars, which are representatives of ancient domestication events [61], or the admixed oleaster genotypes might have been derived from ancient olive cultivars.

The clear separation of native cultivars from foreign cultivars and the presence of oleasters suggest that the most native olive cultivars originated from native oleasters. However, some of the present Albanian olive cultivars were admixed with foreign ones, suggesting that they might be derived from the crossing of native and introduced foreign cultivars (Figure 2A). The differentiation of genotypes according to their regions of origin or main area of distribution, Ionic and Adriatic, was obvious, confirming previous results that were based on morphological characters [1]. Olives are used mainly for oil production or as table olives. Some olives are used for both purposes because they give a good percentage of oil and have large fruit. The lack of differentiation of olive genotypes according to their end-use observed in our results of PcoA and structure analysis (Figure 5) did not support the differentiation of genotypes according to their fruit end-use obtained in UPGMA (Supplementary Figure S1). Analysis of molecular variance confirmed a wide range of variability within groups and significant differences among groups. Our results are in line with previous studies, in which most variation was maintained within olive populations [11,12], suggesting that, especially for wild olive, it is necessary to collect a higher number of samples within sampling sites in future studies [11]. The obtained high level of variance within cultivars may be due to mislabeling and the presence of homonyms in olive germplasm [51].

The exchange of olive plant material that may have occurred between Italy and Albania is also supported by the high similarity observed between some native olive cultivars or oleasters and Italian cultivars grown in Albania, as well as with the cultivars ‘Frantoio’, ‘Leccino’, ‘Carolea’, ‘Ascolana Tenera’, and those from the olive database. The Italian cultivars showed similarity with some native genotypes collected in situ at the central and northern parts of Albania, suggesting that the trade developed in ancient times between these two countries through the port city of Durrësi in Albania, or the well-known trading activities of northern Albania with Venice, indicate how the material exchange took place. Further studies employing a larger number of oleasters, including those of neighboring countries, will elucidate the origin of olive origin in Albania as well as the direction of olive exchange among countries.

A model-based Bayesian approach used to infer the genetic structure of samples resulted in K = 2 clusters as the best fit to the model for olive genetic structuring. The set of genotypes that were assigned to different gene pools showed no clear differentiation based on olive end-use, and while based on their origin, a weak differentiation of Albanian native cultivars was observed, this weak differentiation was also observed in PCoA and AMOVA, where only 7% of variation was observed among native cultivars, oleasters, and foreign cultivars, indicating a mixed genetic structure of the olive genotypes. The low-differentiation structure (K = 2) detected within our analyzed set of samples may be due to complex relationships among the olive cultivars or due to the inclusion of oleasters in the cluster. A weak differentiation of cultivars (51) and wild olives (107) was also reported by Belaj et al. [12], where wild olives and cultivars collected in three main Spanish olive growing regions were divided into four main gene pools. Only in two cases were the majority of wild olives and cultivars coming from the same region, Andalusia and Catalonia, clustered in separate gene pools. The wild olives of Valencia were assigned to another gene pool, while the remaining Andalusian and Valencian cultivated olives were clustered into the fourth gene pool. In contrast, Erre et al. [13] reported the assignment of wild (21) and cultivated olives (57) of Sardinia into two different clusters.

Our population was not structurally based on the end-use of the fruit (oil or table), in contrast to Do Val et al. [47], who reported genetic differentiation to a certain extent according to the end-use of 60 Brazilian olive cultivars, indicating a relationship between their genetic make-up and agronomical traits, such as size of fruit and percentage of the oil. This level of admixture is possibly also due to the make-up of the sample set, in which currently cultivated olive (native and foreign) ancient cultivars and oleaster forms are included.

### 4.5. Core Collection

The conservation of cultivated plants by the establishment of a core collection is essential for the optimal management and use of their genetic resources [18]. Two world germplasm banks, in Spain and in Marrakech, have been constructed, including a huge number of olive cultivars from all over the world. The construction of a core collection is recommended for optimizing an olive germplasm collection. Several studies have arranged core collections of large and important collections. Haouane et al. [18] developed a core collection of 67 olive accessions from a total of 505 accessions from 14 Mediterranean countries planted at OWGB in Marrakech (Morocco). Only 15% (59) of cultivars from the collection of WOGB Córdoba Spain, coming from 21 different Mediterranean countries, were necessary to capture 236 alleles displayed by the WOGB collection [19]. Muzzalupo et al. [21] proposed an Italian olive core collection, capturing all 81 detected alleles of 489 olive cultivars of Italian germplasm CRA-OLI. Only 5% of olive accessions were sufficient to construct the core collection. According to Belaj et al. [20], a core collection containing 10–19% of the total collection size was considered optimal to retain the bulk of genetic diversity found in the IFAPA (Andalusian Institute of Agrarian and Fishing Research and Training), a germplasm collection of 361 olive accessions. In our study, high percentages of accessions (31%) were necessary to capture all the allelic richness found in olive germplasm in Albania, suggesting the presence of high levels of diversity in our sample genotypes. No data was previously reported in core collection development of the olive germplasm in Albania. This information will serve for proper management of collections and genetic resources conservation in the future.

## 5. Conclusions

This study is the most representative analysis of olive germplasm in Albania to date, reporting the existing levels of genetic diversity and structure at a broad national scale. In addition, the present study is the first report on the molecular characterization of Albanian germplasm and an assessment of genetic relationships of oleasters, native cultivars, and imported foreign cultivars. The geographical grouping according to olive origin and area of distribution was confirmed, although not in a clear-cut way. There was weak differentiation of olive genotypes based on their fruit end-use. The analysis of genetic relatedness between native and foreign olive genotypes gives an insight into the origin of Albanian olive cultivars. The native olive genotypes showed tight clustering, suggesting their autochthonous origin. The molecular data provides for the first time a broad inventor of olive genetic resources, as essential information to construct the reference molecular database of Albanian olive germplasm. Herein, we assembled the core collection to represent the entire diversity of the analyzed set of olive genotypes as a valuable source for the development of an improved breeding strategy and for safeguarding the wealth of genetic diversity. The findings will provide a useful resource as well as guidance for better germplasm utilization in genetic improvement and serve as a database for identification and traceability purposes.

## Figures and Tables

**Table 1:** Genetic diversity parameters found in different olive groups/populations: native olive cultivars, oleasters, and imported foreign olive cultivars.

Genetic Diversity Parameters		Cultivars (120)	Oleaster (18)	Foreign (45)
No	Total	180	138	163
No	Mean	6.92	5.31	6.2
	Min	2	2	2
	Max	18	11	14
Na (Freq ≥ 5%)	Mean	3.769	4.269	4.269
Npr	Mean	0.923	0.346	0.500
Ne	Mean	3.28	3.51	3.72
Ho	Mean	0.760	0.745	0.703
	Min	0.403	0.333	0.156
	Max	0.949	1	0.932
He	Mean	0.657	0.692	0.683
	Min	0.323	0.413	0.145
	Max	0.821	0.868	0.899

No—number of alleles, Na—number of alleles with frequency (Freq) ≥ 5%, Ho—observed heterozygosity, Ne—number of effective alleles, Npr—number of private alleles per population, He—uni-based expected heterozygosity = (2N/(2N − 1)) * He. In parenthesis is the number of accessions per each group.

## Data Availability

Nucleotide sequences were deposited with GenBank: SNB 03 (GenBank accession no. KX868599), SNB 11 (GenBank accession no. KX868600), SNB 14 (GenBank accession no. KX868601), SNB 19 (GenBank accession no. KX868602), SNB 20 (GenBank accession no. KX868603), SNB 22 (GenBank accession no. KX868604), SiBi 03 (GenBank accession no. KX868605), SiBi 04 (GenBank accession no. KX868606), SiBi 05 (GenBank accession no. KX868607), SiBi 07 (GenBank accession no. KX868608), SiBi 11 (GenBank accession no. KX868609), and SiBi 19 (GenBank accession no. KX868610).

## Supplementary Materials

The following are available online at https://www.mdpi.com/2073-4425/12/2/256/s1, Figure S1: Dendrogram of 183 olive genotypes based on DICE coefficient and UPGMA clustering method, Figure S2: (A) Mean Log-likelihood (Ln(K) ± SD) averaged over 10 iterations for model-based structure analysis (STRUCTURE), (B) Evanno test for delta K, Table S1: List of 183 unique olive genotypes, their origin, and fruit end-use, Table S2: The genetic relationships of 183 olive genotypes in clusters according to UPGMA dendrogram, Table S3: Pairwise population matrix of Nei’s genetic distance between native olive cultivars, foreign cultivars, and oleasters, Table S4: The representation of Olive genotypes in each group identified by the Bayesian model-cluster method, Table S5: Accessions included in the core collection and their origin.
